# Seizure-induced apnea causes sudden death independent of cortical spike wave discharge in mouse models of Tuberous Sclerosis Complex

**DOI:** 10.1016/j.nbd.2026.107524

**Published:** 2026-07-08

**Authors:** Marissa C. Incer, Ian C. Wenker

**Affiliations:** Department of Anesthesiology, University of Virginia School of Medicine, United States

**Keywords:** SUDEP, TSC, Tonic seizure, Apnea, Seizure, Breathing

## Abstract

Sudden unexpected death in epilepsy (SUDEP) is the leading cause of seizure-related mortality for people with epilepsy (PWE). Current evidence suggests that many cases of SUDEP are due to seizures that produce respiratory arrest, aka seizure-induced apnea. Patients with tuberous sclerosis complex (TSC) are at a significant risk of SUDEP, and mouse models of TSC have early mortality. Previous reports in TSC mouse models demonstrate that although Vigabatrin suppresses seizures, it does not improve survival. In other mouse models, our team has demonstrated that seizure-induced apnea occurs during tonic seizures, and that cortical spike-wave discharge (SWD) is not necessary to produce apnea during exogenously stimulated seizures. We hypothesized that seizure-induced apnea drives fatality independent of SWD in the spontaneous seizures of TSC mouse models, and this is why Vigabatrin treatment is ineffective. Two TSC mouse models were continuously monitored with electroencephalography, electrocardiography, plethysmography, and video recording to detect spontaneous seizures and SUDEP events. A subset of mice received daily Vigabatrin. Both TSC mouse models exhibited two spontaneous seizure types: clonic seizures with SWD and tonic seizures without SWD. All seizure-related fatalities were due to seizure-induced apnea. Across 21 SUDEP events, 20 occurred immediately after tonic seizures. Vigabatrin nearly eliminated clonic seizures but did not prevent tonic seizures and the concurrent apnea. In addition, Vigabatrin-treated mice exhibited cardiac signals that declined hours before SUDEP. These findings underscore the need for SUDEP-specific interventions beyond seizure suppression.

## 1. Introduction

Sudden unexpected death in epilepsy (SUDEP) is defined as the sudden death of a person with epilepsy that is not due to *status epilepticus*, nor does a postmortem examination reveal another cause of death (Nashef et al., 2012). SUDEP is responsible for a significant portion of all epilepsy-related deaths, ranging from 8 to 17%, which escalates to 50% for patients with refractory seizures (Devinsky, 2011; Tolstykh and Cavazos, 2013). The limited data available for clinical SUDEP suggest that respiratory arrest, secondary to a convulsive seizure, is the primary final culprit of SUDEP (Ryvlin et al., 2013). Obtaining detailed clinical data is prohibitive; however, in mouse models of SUDEP, our group and others have found that respiratory arrest secondary to seizures, aka seizure-induced apnea, is the ultimate cause of death.

Tuberous sclerosis complex (TSC) is a genetic disorder caused by pathogenic variants in either TSC1 or TSC2. TSC patients experience clinical variability; however, seizures are one of the most common and challenging symptoms, occurring in up to 85–90% of affected individuals. Unfortunately, early mortality for patients with Tuberous Sclerosis Complex (TSC) is high (Zöllner et al., 2020). Although this is due to a variety of causes (Zöllner et al., 2020), recent reports indicate that 22 to 35% of early mortality is due to SUDEP (Amin et al., 2017; Parthasarathy et al., 2021; Pentz et al., 2025).

Mouse models with loss-of-function TSC alleles produce many of the hallmarks seen in patients, including convulsive seizures and premature mortality (Meikle et al., 2007; Zhang et al., 2013). The cause of this premature mortality has not been explored. Interestingly, Vigabatrin, a GABA transaminase inhibitor used clinically in TSC patients (Curatolo et al., 2001), has been shown to essentially eliminate seizures in TSC model mice (Zhang et al., 2013). However, Vigabatrin had minimal impact on their survival. This suggests that while Vigabatrin may block certain types of clearly visible seizures (i.e., those with spike-wave discharge (SWD)), it may have minimal effect on tonic seizures, which are more likely to present with less noticeable fast paroxysmal activity.

Our group has found in mouse models of *Scn8a* epilepsy that seizure-induced apnea is coincident with the tonic phase of seizures (Wengert et al., 2021; Wenker et al., 2021, 2022a). This apnea that occurs during the tonic phase (aka, tonic phase apnea) appears to be due to the tonic contraction of respiratory muscles (Liška et al., 2024; Umezu et al., 2024; Wenker et al., 2021). We have also observed that cortical SWD often occurs after the tonic phase in our mice (Wengert et al., 2021), suggesting that SWDs are not necessary for the production of the behavioral tonic phase apnea. To test whether SWDs are required for tonic phase apnea directly, we inhibited SWDs using a chemogenetic approach targeted to the forebrain during audiogenic seizures, and found that tonic phase apnea persists (Wenker et al., 2022b). This has led to our overarching hypothesis that the neural circuitry underlying this tonic phase apnea resides in the brainstem. However, whether this lack of SWD dependency occurs in spontaneous seizures is an open question.

We hypothesized that in TSC models, terminal apnea is initiated during the tonic phase of seizures, and that although Vigabatrin can block SWDs, it does not affect the incidence of tonic phase apnea. We chronically recorded breathing, cardiac, and video/EEG activity up to and during SUDEP in two of the most commonly used TSC mouse models: Tsc1^GFAP-Cre^ and Tsc1^Syn1-Cre^ mice. In a subset of Tsc1^GFAP-Cre^ mice, we administered Vigabatrin daily to assess its effects on seizures and mortality. As we predicted, in both strains, tonic seizures produced seizure-induced apnea and fatality. We found that clonic seizures were nearly eliminated by Vigabatrin; however, tonic seizures persisted and caused mortality.

## 2. Results

### 2.1. Nonfatal clonic and tonic seizures observed in Tsc1^GFAP-Cre^ mice

Tsc1^GFAP-Cre^ mice experienced convulsive seizures, which were classified into two groups: clonic seizures that presented electrographically with spike wave discharge (SWD) and tonic seizures that presented electrographically with paroxysmal fast activity (PFA). During clonic seizures (Fig. 1B), breathing and cardiac activity were either normal or elevated (Fig. 1C and D). Tonic seizures did not present with SWDs but were identified based on 1) the electrographic signatures of bleed-through of muscle activity into the ECG signal (Fig. 2A) and PFA in the EEG signal (Fig. 2B & C), and 2) the behavioral signature of hindlimb extension (not shown). In addition, apnea was coincident with the tonic phase (Fig. 2A), as we have observed in other mouse models (Wengert et al., 2021; Wenker et al., 2021, 2022b).

In the 48 h prior to death, a total of 39 clonic and four tonic nonfatal seizures were observed in the 5 Tsc1^GFAP-Cre^ mice (mean of 8.6 seizures per mouse). In addition to being more common, clonic seizures were significantly longer than tonic seizures: 94.4 ± 11.7 s compared to 26.3 ± 1.7 s, respectively (Fig. 2D; all statistical tests are reported in the figure legends). Due to the presence of SWD, clonic seizures produced a significant increase in EEG power (examples in Supp. Fig. 1) from preictal 0.33 ± 0.03 mV^2^ to ictal 1.93 ± 0.18 mV^2^, while this was not observed in tonic seizures (0.22 ± 0.01 mV^2^ to 0.11 ± 0.01 mV^2^, respectively; Fig. 2E). Finally, both clonic and tonic seizures experienced an increase in the mean frequency of the FFT of the EEG signal, which was larger in the tonic seizures (Fig. 2F).

### 2.2. Fatal seizures in Tsc1^GFAP-Cre^ mice

Eight fatal seizures were recorded in Tsc1^GFAP-Cre^ mice. Seven of the fatal seizures had a clear tonic phase, and only one did not. In comparison, only one of 39 clonic seizures was fatal (Fig. 3A). In addition, all fatal seizures were due to terminal apnea after a seizure, minutes prior to terminal asystole (Fig. 3B). For the seven tonic seizures, terminal apnea was initiated with the tonic phase (Fig. 3C). For the single fatal non-tonic seizure, terminal apnea began not long after a clonic seizure ended (Supp. Fig. 2). Three of the seven fatal tonic seizures presented with proximal SWD (example shown in 3C); however, in these cases the tonic seizure immediately followed a series of clonic seizures which had SWDs.

### 2.3. Vigabatrin suppresses clonic but not tonic seizures in Tsc1^GFAP-Cre^ mice

Vigabatrin (VGB) is a first-line therapy for treating epileptic spasms in children with TSC (Curatolo et al., 2001) and has been shown to reduce seizures in Tsc1^GFAP-Cre^ mice. In a previous report (Zhang et al., 2013) VGB profoundly inhibited seizures in Tsc1^GFAP-Cre^ mice, but only produced a ~ 3-week increase in survival when administered at a dose of 200 mg/kg/day. Using the same dosage, we obtained similar results. The VGB-treated Tsc1^GFAP-Cre^ mice survived ~3 weeks longer than their untreated counterparts: 10.5 ± 0.7 and 7.4 ± 0.3, respectively (Fig. 4A). In addition, VGB-treated mice experienced significantly less clonic seizures than untreated mice (Fig. 4B). However, we also found that nonfatal tonic seizures were not significantly affected by VGB treatment (Fig. 4C). Although the VGB-treated mice lived longer, tonic seizures were still the mechanism of fatality in this group of mice (Fig. 4D), just as they were in untreated Tsc1^GFAP-Cre^ mice (Fig. 3C).

### 2.4. Fatal seizures in Tsc1^Syn1-Cre^ mice

We also explored the cause of mortality in another TSC mouse model: Tsc1^Syn1-Cre^ mice (Fig. 5A). The survival curves of the untreated Tsc1^Syn1-Cre^ mice and Tsc1^GFAP-Cre^ mice were similar to previous reports (Meikle et al., 2007; Zeng et al., 2008), with age at death being 4.4 ± 0.6 and 7.4 ± 0.8 weeks, respectively (Fig. 5B). All observed deaths in Tsc1^Syn1-Cre^ mice occurred after the onset of tonic seizures (Fig. 5C). No SWD was observed during fatal seizures in Tsc1^Syn1-Cre^ mice.

Tsc1^Syn1-Cre^ mice exhibit a more severe phenotype than Tsc1^GFAP-Cre^ mice; they are smaller and exhibit behavioral problems in addition to seizures (Meikle et al., 2007). We found their breathing signal to be small, but measurable, at first; however, by the time of the fatal seizures, breathing was impaired to the point that it could not be detected above background noise (Fig. 5E and F). Thus, whether the tonic phase coincided with apnea, as we have previously reported in other SUDEP mouse models, could not be determined. However, the same sequence of events occurred as with other models and the Tsc1^GFAP-Cre^ mice. In both TSC models, terminal apnea (assessed as the tonic phase in Tsc1^Syn1-Cre^ mice) preceded terminal asystole by several minutes: 6.4 ± 1.6 and 7.1 ± 2.2 min for Tsc1^Syn1-Cre^ mice and Tsc1^GFAP-Cre^ mice, respectively (Supp. Fig. 3).

### 2.5. Heart and breathing rate decrease prior to fatal seizures in VGB-treated Tsc1^GFAP-Cre^ mice and untreated Tsc1^Syn1-Cre^ mice

It was noted in both Tsc1^Syn1-Cre^ mice and VGB-treated Tsc1^GFAP-Cre^ mice that their breathing and cardiac signals were diminished prior to the fatal seizures (Fig. 4F compared to 4E and Fig. 5E compared to 5D). In contrast, respiratory and cardiac signals appeared normal for fatal seizures in untreated Tsc1^GFAP-Cre^ mice (Fig. 3D). Therefore, we compared recorded vital signs in VGB-treated and untreated Tsc1^GFAP-Cre^ mice over the 10 h before the fatal seizure. Treated and untreated Tsc1^GFAP-Cre^ mice had similar heart rates (HR) at 10 h before the seizure (Fig. 6A). However, the HR of the mice treated with VGB decreased prior to the fatal seizure, leading to a significantly lower HR prior to the seizure compared to the nontreated mice (Fig. 6B). Similar results were found for breathing amplitude. Breathing amplitude was similar for treated and untreated mice 10 h before the fatal seizure (Fig. 6C), but decreased to a significantly lower amplitude in the VGB-treated mice compared to the untreated mice (Fig. 6D). Although formal analysis was not performed, VGB-treated Tsc1^GFAP-Cre^ mice were not observed to behave any differently than their untreated counterparts; they groomed and ate normally.

## 3. Discussion

### 3.1. Summary of results

In the present study, we show that 1) Tsc1^GFAP-Cre^ and Tsc1^Syn1-Cre^ mice experience two types of seizures with mutually exclusive semiology: clonic seizures that present with cortical SWD and tonic seizures that lack SWD and present with tonic phase apnea; 2) 20 out of 21 of the observed SUDEP events were due to tonic phase apnea; 3) daily administration of VGB blocked clonic seizures, but not tonic seizures; 4) while VGB-treated animals lived longer, tonic seizures were still the perpetrator of early mortality; and 5) VGB-treated Tsc1^GFAP-Cre^ mice and Tsc1^Syn1-Cre^ mice showed decreased heart rate and EEG power hours prior to death, whereas untreated Tsc1^GFAP-Cre^ mice did not.

### 3.2. Mechanism of death in SUDEP mouse models

We have previously shown in mouse models harboring patient-derived pathogenic variants of *Scn8a* that spontaneous seizure-induced death (aka, SUDEP) occurs exclusively from respiratory arrest proximal to a seizure (Wenker et al., 2021). Furthermore, the fatal seizures in these mice were always dominated by a prominent tonic phase, which can be observed physiologically as muscle activity recorded in the ECG lead and behaviorally as hindlimb extension. In other mouse models, including Scn1a^R1407X^, DBA1/2, Kcna1^−/−^ , 129/SvTer, and Cacna1a^S218L^ mice, fatal seizures are classified as tonic, generally based on the observation of hindlimb extension (Dhaibar et al., 2019; Faingold et al., 2010, 2016; Jansen et al., 2019; Kim et al., 2018; Loonen et al., 2019; Martin et al., 2020). The present study fits squarely with these previous results.

While our observations of tonic phase apnea appear common across many mouse models, it is not universal. For instance, in a mouse model of Dravet Syndrome, a subset of fatal heat-induced seizures was described as clonic (Kim et al., 2018). In addition, results in rat models demonstrate more variability in fatal seizure type. In one study using high-dose pentylenetetrazole (PTZ) injection into rats, all fatal seizures were found to include a tonic phase (Umezu et al., 2024); however, another study found that PTZ-induced seizures in rats produced fatal seizures where most entirely lacked a tonic phase (Liška et al., 2024). In mice, we observed that PTZ-induced fatal seizures were all of the tonic variety (Wenker et al., 2021). In the present study, we did find a single fatal seizure that did not have a detectable tonic phase.

Thus, tonic phase apnea is a common cause of death in rodent SUDEP models. Our current understanding of tonic phase apnea does not preclude that the tonic phase and the apnea are coincident with, but not dependent on, one another. However, as of yet, there is no method to eliminate or inhibit the tonic phase of seizures experimentally. Further research on the circuitry underlying the tonic phase and breathing cessation may help us disambiguate the two.

### 3.3. Mechanisms of the tonic phase of seizures

Seizures are generally considered a forebrain phenomenon. However, even decades ago, it was recognized that seizures may originate in the brainstem (Nathanson, 1978), and seizure activity spreading to brainstem areas has been documented in animal models (Lertwittayanon et al., 2020). The tonic phase of seizures has been linked to subcortical activity. Early studies investigating SUDEP used genetically epilepsy-prone rats (GEPRs) to demonstrate hyperexcitability in the inferior colliculus, periaqueductal gray, and pons (Faingold et al., 2017). These studies use audio-induced seizures, which may be different and less clinically relevant than other seizures; however, the seizure behaviors are nearly identical to seizures initiated in the temporal lobe (Hirsch et al., 1992), suggesting a common circuit mechanism.

Previously, we tested this by expressing synthetic GiDREADD receptors (Roth, 2016) in forebrain excitatory neurons, allowing us to suppress cortical SWDs during audio- and temporal lobe-stimulated seizures selectively (Wenker et al., 2022b). In both cases, tonic phase apnea persisted in the absence of SWDs, indicating a lack of dependence (Wenker et al., 2022b). These previous experiments only addressed stimulated seizures. In the present study, we found that tonic seizures occurred without SWDs. It was also observed that the tonic seizures occurred with PFA in the EEG signal, as is reported for clinical tonic seizures (Bhatt et al., 2011). In Lennox-Gastaut patients experiencing clonic and tonic seizures, fMRI indicates that diffuse brain activity and simultaneous activation of subcortical regions, including the thalamus, basal ganglia, and brainstem, occur during PFA (Bhatt et al., 2011). Taken together, this indicates that the neural circuitry that drives fatal apnea and SUDEP may be very different than what is commonly associated with generalized seizures and likely includes the brainstem.

### 3.4. Seizure frequency and SUDEP occurrence

SUDEP risk in patients is roughly proportional to convulsive seizure frequency (Jha et al., 2021), and those with refractory epilepsy are at the highest risk (Tolstykh and Cavazos, 2013). In other words, the more seizures a person has, the more likely that person is to die of SUDEP. However, this correlation is rough, and counterexamples exist; perhaps the most famous being that of Cameron Boyce, the child actor who died after having only a few seizures.

In the present study, we found that VGB treatment nearly eliminated clonic seizures. Interestingly, although VGB reduced seizures, survival only increased marginally. Furthermore, the mechanism of death was identical to that of the untreated mice: i.e., apnea initiated during the tonic phase. As described above, the tonic seizures were not affected by VGB. Our findings fit with the previous observation that similar dosages of VGB could reduce clonic, but not tonic, phases of seizures have also been reported in audiogenic rat models (Vinogradova et al., 2005). However, if the fatal type of seizure is unaffected by VGB, why was survival increased at all?

One interpretation is that although clonic seizures do not impair breathing or result in fatality, they may alter the neural networks to make tonic seizures more likely to occur or to be fatal. Interestingly, the VGB-treated Tsc1^GFAP-Cre^ mice exhibited a progressive impairment of breathing and cardiac function in the 5–10 h preceding SUDEP. One interpretation is that VGB somehow impairs these systems; however, we observe the same phenomenon in untreated Tsc1^Syn1-Cre^ mice. In addition, the VGB treatment often lasted for several weeks before the cardiorespiratory impairment became apparent close to death. This suggests the decrease in heart rate and breathing was not due to VGB itself. It is also possible that continued lack of the TSC1 gene product causes other effects besides seizures, and eventually this results in impaired cardiorespiratory control that can precipitate SUDEP. Most likely, the Tsc1^Syn1-Cre^ mice have greater Tsc1 reduction in neurons of the brainstem, compared to Tsc1^GFAP-Cre^ mice. This may contribute to the earlier onset of progressive cardiorespiratory decline in Tsc1Syn1-Cre mice. Given the extended lifespan conferred by VGB administration, the Tsc1GFAP-Cre mice may eventually succumb to the same impairment resulting from Tsc1 reduction. Selective KO of Tsc1 in forebrain neurons is certainly sufficient to produce early mortality (Carson et al., 2012), but brainstem expression may further exacerbate this problem. Further examination of Tsc1 deletion in the brainstem would be warranted.

## 4. Materials and methods

### 4.1. Mouse husbandry and genotyping

All mice were housed and cared for in accordance with NIH guidelines in a 12-h light/dark cycle with food and water ad libitum. All procedures were carried out at the University of Virginia and approved by the Animal Care and Use Committee. The breeding scheme to produce experimental mice is depicted in Figs. 1A and 5A. Mice with a conditional Tsc1 allele (“fl-Tsc1”, Jax # 038428) were crossed either with GFAP-Cre (provided by Psychogenics, Inc., and the same as Jax # 004600) or Syn1-Cre (Jax # 003966) mice. Male mice homozygous for the conditional Tsc1 allele (fl-Tsc1/fl-Tsc1) were crossed with female mice heterozygous for the conditional Tsc1 allele (fl-Tsc1/+) and the Cre allele (Cre+/− ). Experimental mice were heterozygous for Cre and homozygous for the conditional Tsc1 allele: i.e., Syn1-Cre(+/− );fl-Tsc1/fl-Tsc1 (referred to as Tsc1^Syn1-Cre^) and GFAP-Cre(+/− );fl-Tsc1/fl-Tsc1 (referred to as Tsc1^GFAP-Cre^). Genotyping was performed to confirm the presence of the fl-Tsc1 and Cre alleles by Transnetyx, Inc. (Cordova, TN, United States. A total of 11 Tsc1^Syn1-Cre^ and 23 Tsc1^Syn1-Cre^ mice were used. Male and female mice were used with no observable difference in seizure phenotype or mortality.

### 4.2. Surgical preparation

Custom electrocorticogram (EEG)/electrocardiogram (ECG) headsets were implanted in 4-week-old Tsc1^Syn1-Cre^ and 5 to 7-week-old Tsc1^GFAP-Cre^ mice using standard aseptic surgical techniques, as done previously (Wengert et al., 2021; Wenker et al., 2021, 2022a). Anesthesia was induced with 5% and maintained with 0.5–3% isoflurane. Adequacy of anesthesia was assessed by the lack of toe-pinch reflex. A midline skin incision was made over the skull, and the skull was cleared of connective tissue with 3% peroxide. Burr holes were drilled in both the left and right frontal bones to place EEG leads in the motor cortex at the approximate coordinates of 1 mm rostral, 1 mm lateral, and 1.5 mm ventral to bregma. Burr holes were also drilled in the occipital bone for reference and ground electrodes. A single ECG lead was passed subcutaneously to the left abdomen and sutured into place. Mice received post-operative analgesia with ketoprofen (5 mg/kg, i.p.) and 0.9% saline (0.5 ml, i.p.) and were allowed to recover in their home cage for at least 3 days before experiments.

### 4.3. Recording of electroencephalogram, electrocardiogram, and breathing

Recording of EEG, ECG, and breathing was performed as previously described (Wengert et al., 2021; Wenker et al., 2022b). Plethysmography chambers were built to comply with requirements for continuous housing described in the Guide for the Care and Use of Laboratory Animals (Council, 2011). The floor of the chambers had approximate dimensions of 4.5 × 4.5 in. (>20 sq. inches) and a height of 7 in. There were ports for air in and air out, and for pressure monitoring. The chamber was supplied with a continuous flow of room air at approximately 400 ml/min via supply and exhaust air pumps (MK-1504 Aquarium Air Pump; AQUA Culture) balanced to maintain chamber pressure near atmospheric. Mice had access to a continuous supply of water and food. The surgically implanted headsets were attached to a custom low-torque swivel cable, allowing mice to move freely in the chamber. To assess breathing frequency, the pressure of the EMU chamber was measured with an analog pressure transducer (Validyne Engineering, Canoga Park, California, United States). Electroencephalogram (EEG) and electrocardiogram (ECG) signals were amplified at 2000 and bandpass-filtered between 0.3–100 and 30–300 Hz, respectively, using an analog amplifier (Model 3600, A-M Systems, Sequim, Washington, United States). Biosignals were digitized with a Micro1401–4 at 1000 samples/s, and recorded using Spike2 software (Cambridge Electronic Design, Ltd., Cambridge, United Kingdom). Video was acquired with USB-driven webcams equipped with integrated night vision and recorded at 30 fps using Spike2 software, in tandem with biosignals.

### 4.4. Vigabatrin treatment

Vigabatrin (VGB) was obtained from Millipore-Sigma (St. Louis, Missouri, United States), dissolved in sterile water to a concentration of 40 mg/ml, sterile-filtered, and stored at −20 ^◦^C. Fourteen Tsc1^GFAP-Cre^ mice received i.p. Vigabatrin (VGB, 200 mg/kg) treatment once daily, starting at five weeks of age and continuing until death, as previously reported, to eliminate seizures in this mouse model (Zhang et al., 2013). Mice were injected between 15:00 and 17:00 every day, alternating between left and right abdomens to avoid tissue damage.

### 4.5. Biosignal analysis

In vivo biosignal data were analyzed with Spike2 software (Cambridge Electronic Design, Ltd., Cambridge, United Kingdom). Determination of seizure frequency (Fig. 4), heart rate (HR), plethysmography amplitude, and EEG power (Fig. 6) was done by an experimenter blinded to the treatment at the time of analysis. HR was determined by integrating the ECG signal, smoothing it, and using the peak-finding function in Spike2. The experimenter validated the detected events, which were binned every 60 s and are reported as beats per minute (BPM). Plethysmography amplitude was determined by removing the DC component of the signal, calculating the RMS amplitude at each time point, and then binning the data every 60 s. EEG power was performed with Spike2 software's built-in Fast Fourier Transformation (FFT) using a Hanning window with a window size of 1024 data points (1.024 s), resulting in a frequency resolution of 0.9766 Hz, and binned every 60 s. Apnea imitation and duration were estimated based on the plethysmography signal. Often, wild running immediately prior to the tonic phase can introduce noise into the plethysmography signal, but the initiation of apnea is still accurate within 1–2 s, which is sufficient for the current study.

### 4.6. Statistical analysis

All data points represent biological replicates (i.e., no animal was used more than once for the same test), except for Fig. 2D-F, which represent individual seizures. All average data values are expressed as mean ± SEM, unless otherwise noted. Descriptive statistics and statistical tests were computed using GraphPad Prism version 9 (GraphPad Software, Inc., San Diego, CA, United States). All statistical tests are reported in the figure legends. Comparisons were considered statistically detectable when *p* < 0.05. Comparison between two groups was performed with a nonparametric Mann-Whitney test, except for: survival curves that were compared with the Mantel-Cox test; categorical variables that were tested with Fisher's Exact test; and seizure frequency that was tested with Poisson regression with drug treatment as the explanatory variable. Comparisons between more than two groups were performed using ANOVA and appropriate multiple-comparison tests.

## Supplementary Material

MMC1

## Figures and Tables

**Fig. 1.**
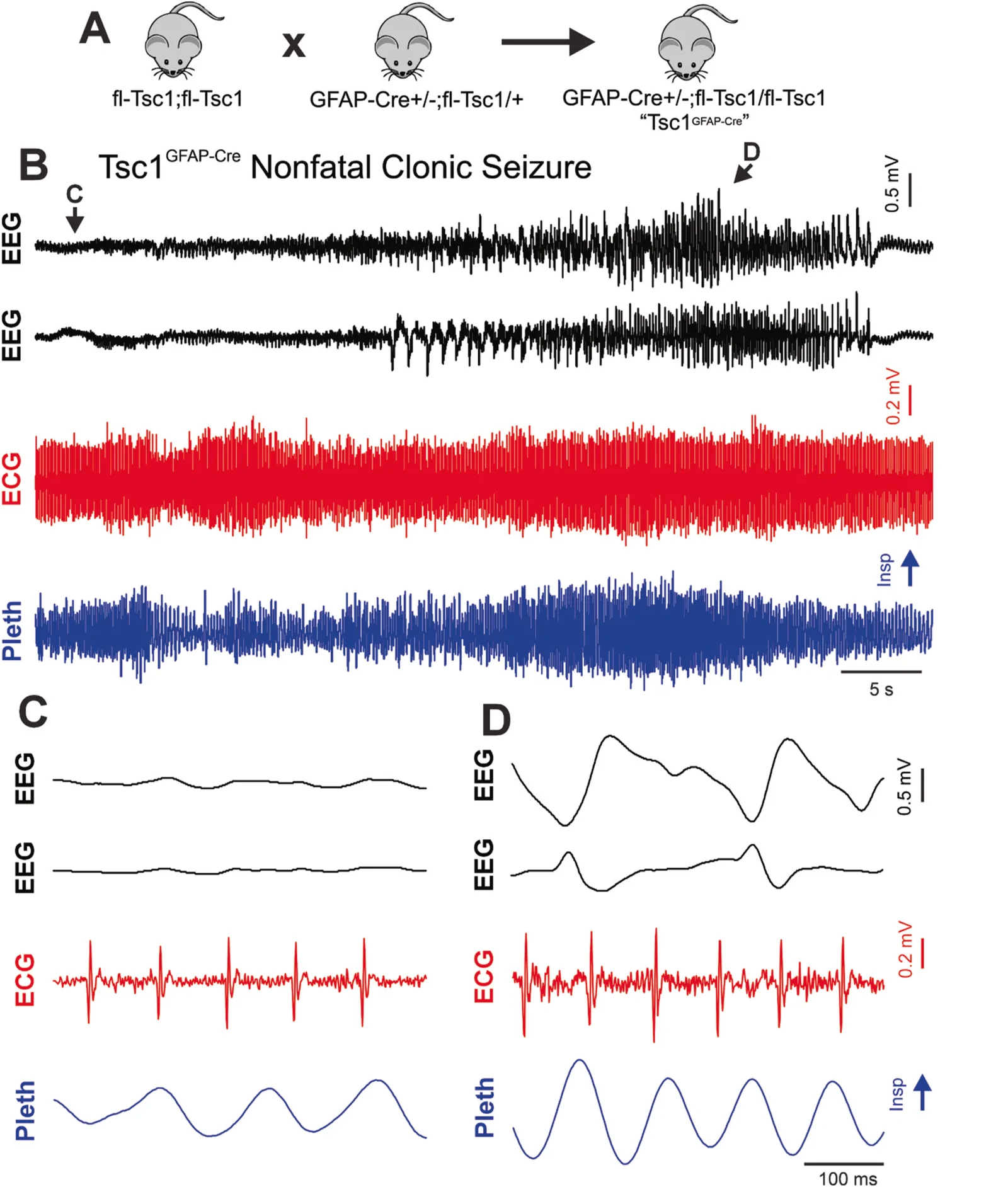
Nonfatal clonic seizures in Tsc1^GFAP-Cre^ mice. A, Breeding strategy to produce TSC mouse model. B, An example of an EEG, ECG, and breathing (pleth) recording from a Tsc1^GFAP-Cre^ mouse shows SWD without apnea or a tonic phase. C–D, Expanded signals from preictal (C), and ictal (D) phases. Note SWD and lack of apnea.

**Fig. 2.**
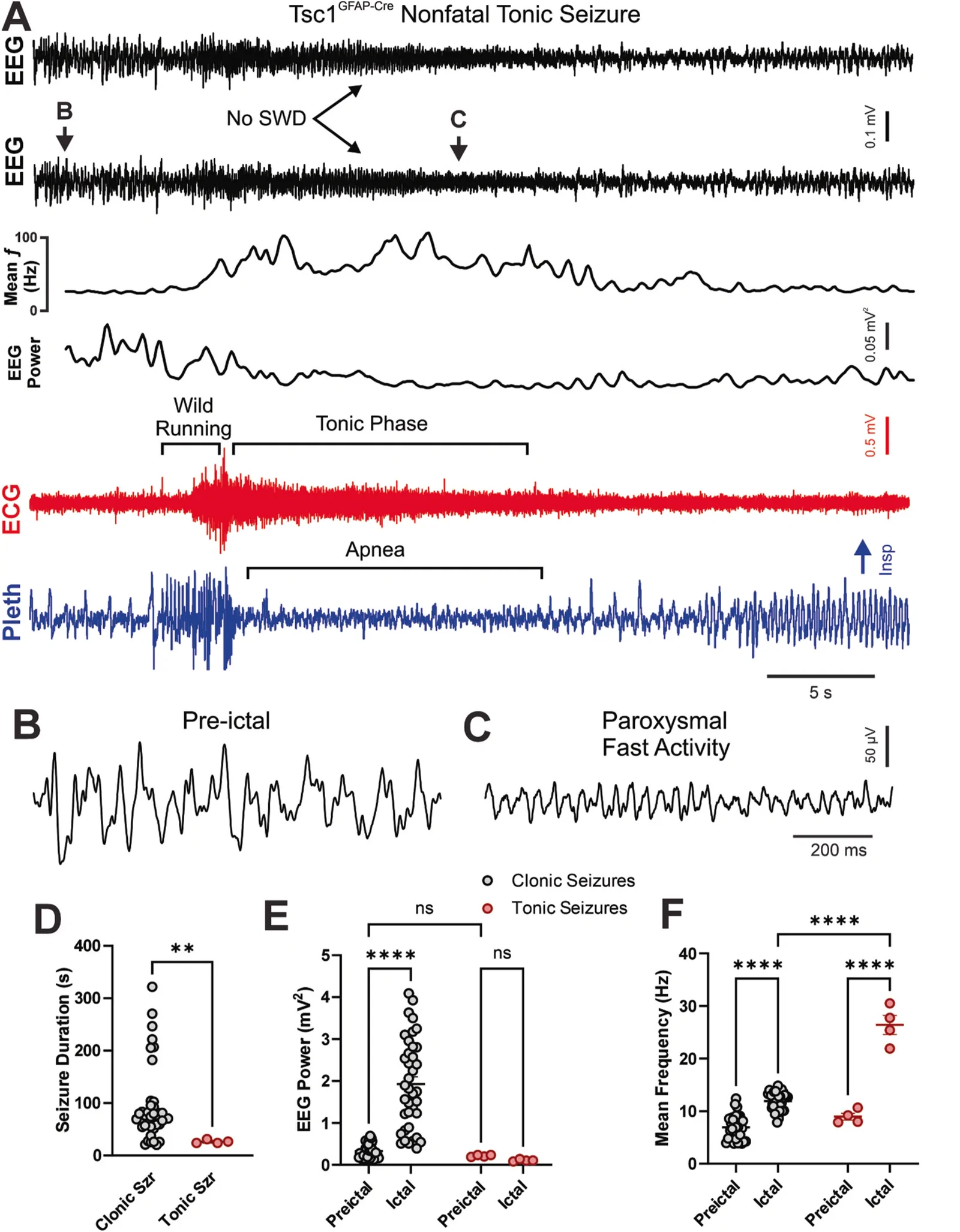
Tsc1^GFAP-Cre^ experience nonfatal tonic seizures without SWDs. A, Example of EEG, derived EEG mean frequency, derived EEG power, ECG, and breathing (pleth) recording from a Tsc1^GFAP-Cre^ mouse during a nonfatal tonic seizure. B and C, EEG traces demonstrating low frequency oscillations before (B), and during (C), the seizure. D, Clonic seizures (black) were significantly longer than tonic seizures (red; *p* = 0.0012, Mann-Whitney test). E, Comparison of EEG power before and during clonic (black) and tonic (red) seizures. EEG power increases significantly for clonic seizures but does not for tonic seizures (*p* < 0.0046 and > 0.9999, respectively, Sidak's multiple comparison test after two-way ANOVA significant for seizure type, ictal/preictal, and interaction factors, F_1,80_ = 9.611, 7.294, and 12.21, respectively, and *p* = 0.0.008, 0.0084, and 0.0027, respectively). F, Comparison of EEG mean frequency before and after clonic (black) and tonic (red) seizures. Mean frequency increases for both clonic and tonic seizures (*p* < 0.0001 for both comparisons, Tukey's multiple comparison test after two-way ANOVA significant for ictal/preictal factor, p < 0.0001); however, mean frequency is higher in tonic compared to clonic seizures (p < 0.0001, Tukey's multiple comparison after two-way ANOVA significant for seizure type factor, F_1,72_ = 108.4 and p < 0.0001). (For interpretation of the references to colour in this figure legend, the reader is referred to the web version of this article.)

**Fig. 3.**
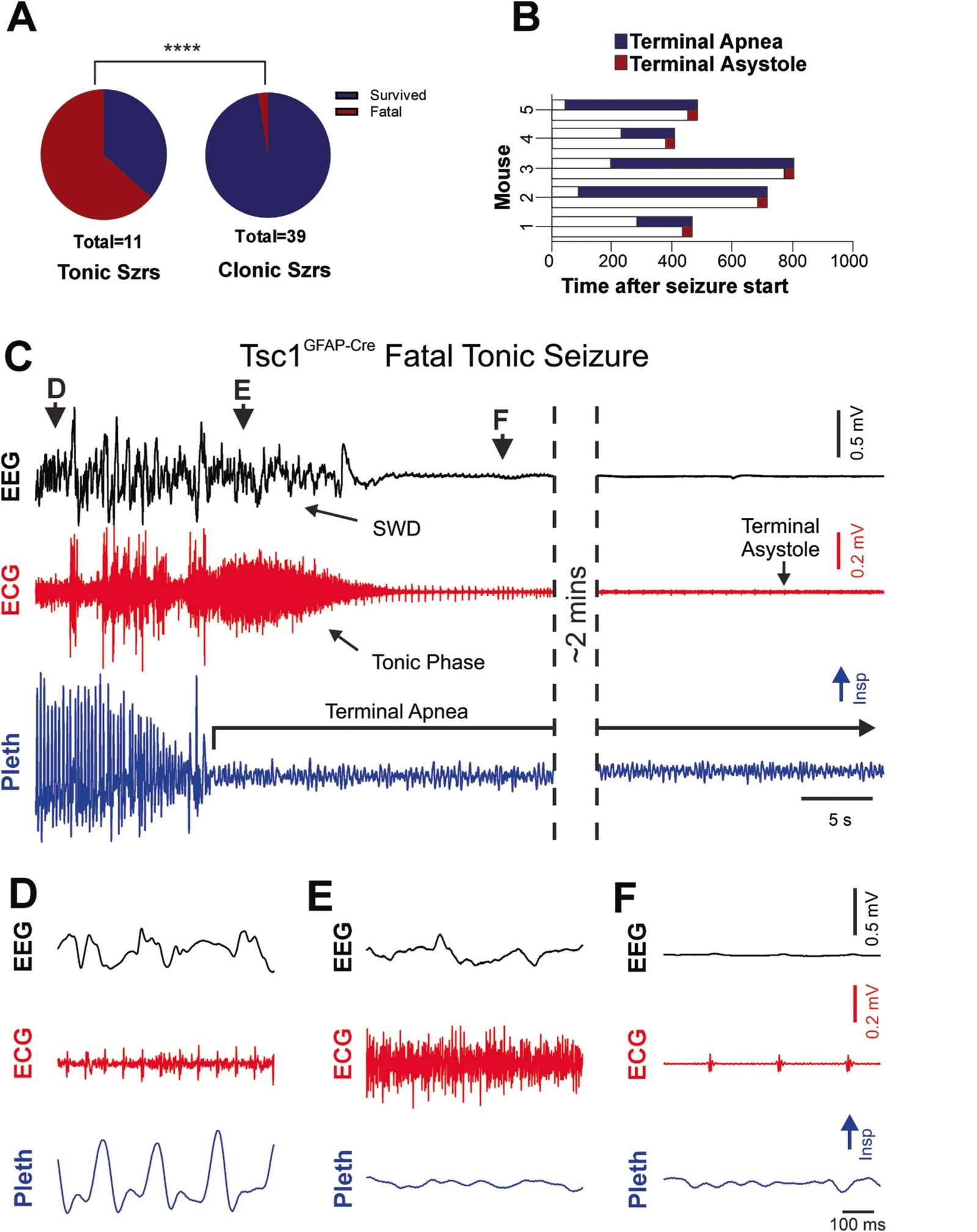
Tonic seizures are fatal in Tsc1^GFAP-Cre^ mice. A, Seven of 11 tonic seizures were fatal compared to 1 of 39 clonic seizures (p < 0.0001, Two-sided Fisher's Exact test). B, All recorded fatalities in Tsc1^GFAP-Cre^ mice had terminal apnea that preceded terminal asystole. C, Example of EEG, ECG, and breathing (pleth) recording from a Tsc1^GFAP-Cre^ mouse during and after a fatal seizure. D–F, Expanded signals from preictal (D), and immediately before (E) and during (F) the tonic phase of the fatal seizure.

**Fig. 4.**
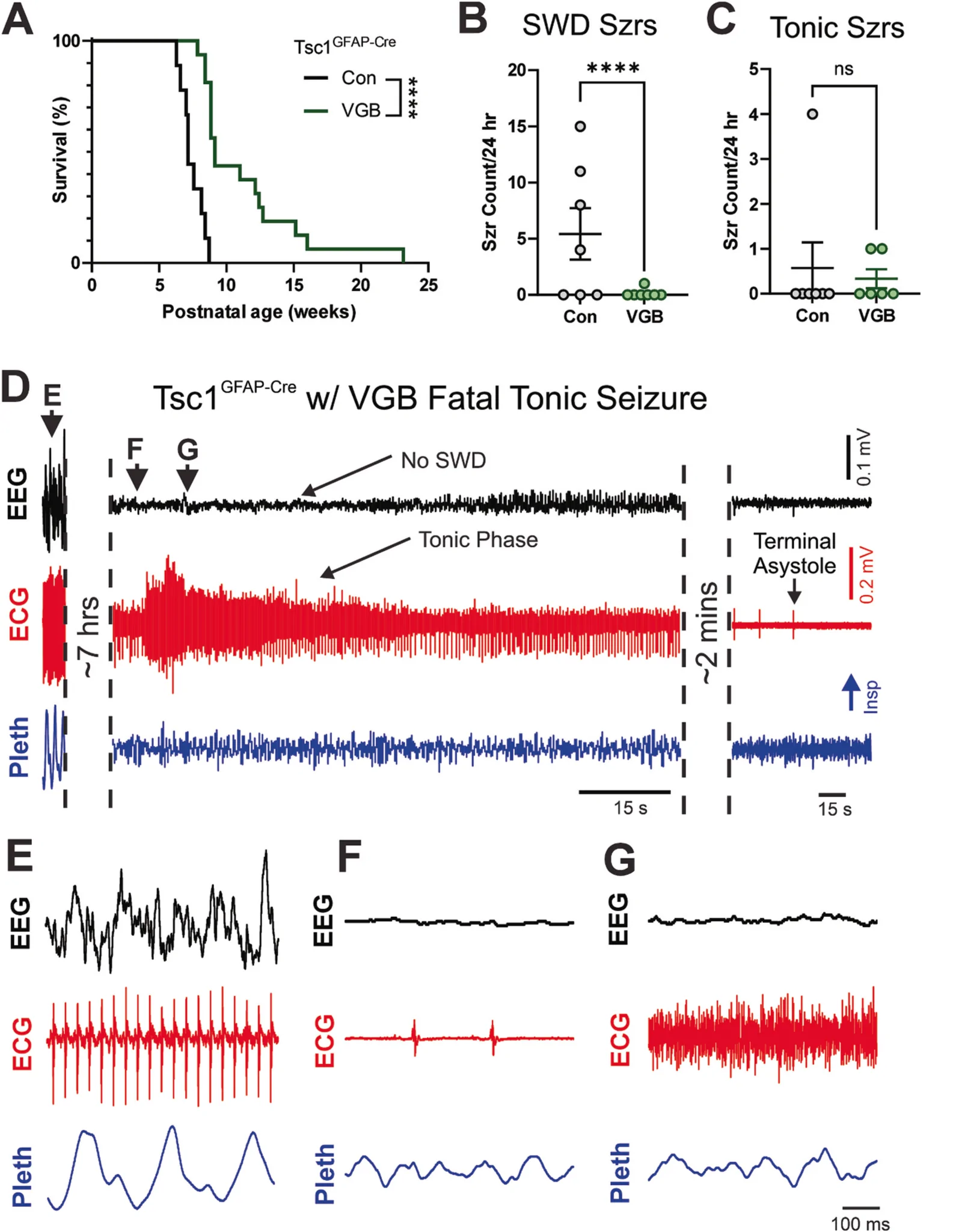
Mice treated with Vigabatrin live longer but still die from fatal tonic seizures. A, Daily administration of Vigabatrin (VGB, 200 mg/kg daily) to Tsc1^GFAP-Cre^ mice resulted in prolonged survival (p < 0.0001, Mantel-Cox test). B–C, VGB treatment significantly reduced clonic seizure frequency (B, p < 0.0001; Poisson regression with drug treatment as an explanatory variable) but not tonic seizure frequency (C, *p* = 0.5235; Poisson regression with drug treatment as an explanatory variable). D, Example of EEG, ECG, and breathing (pleth) recording from a Tsc1^GFAP-Cre^ mouse treated with VGB 24 h before (left of dotted lines) and during (right of dotted lines) a fatal seizure. Not the breathing signal has degraded before the fatal seizure. E-G, Expanded signals from preictal (E), and immediately before (F) and during (G) the tonic phase of the fatal seizure.

**Fig. 5.**
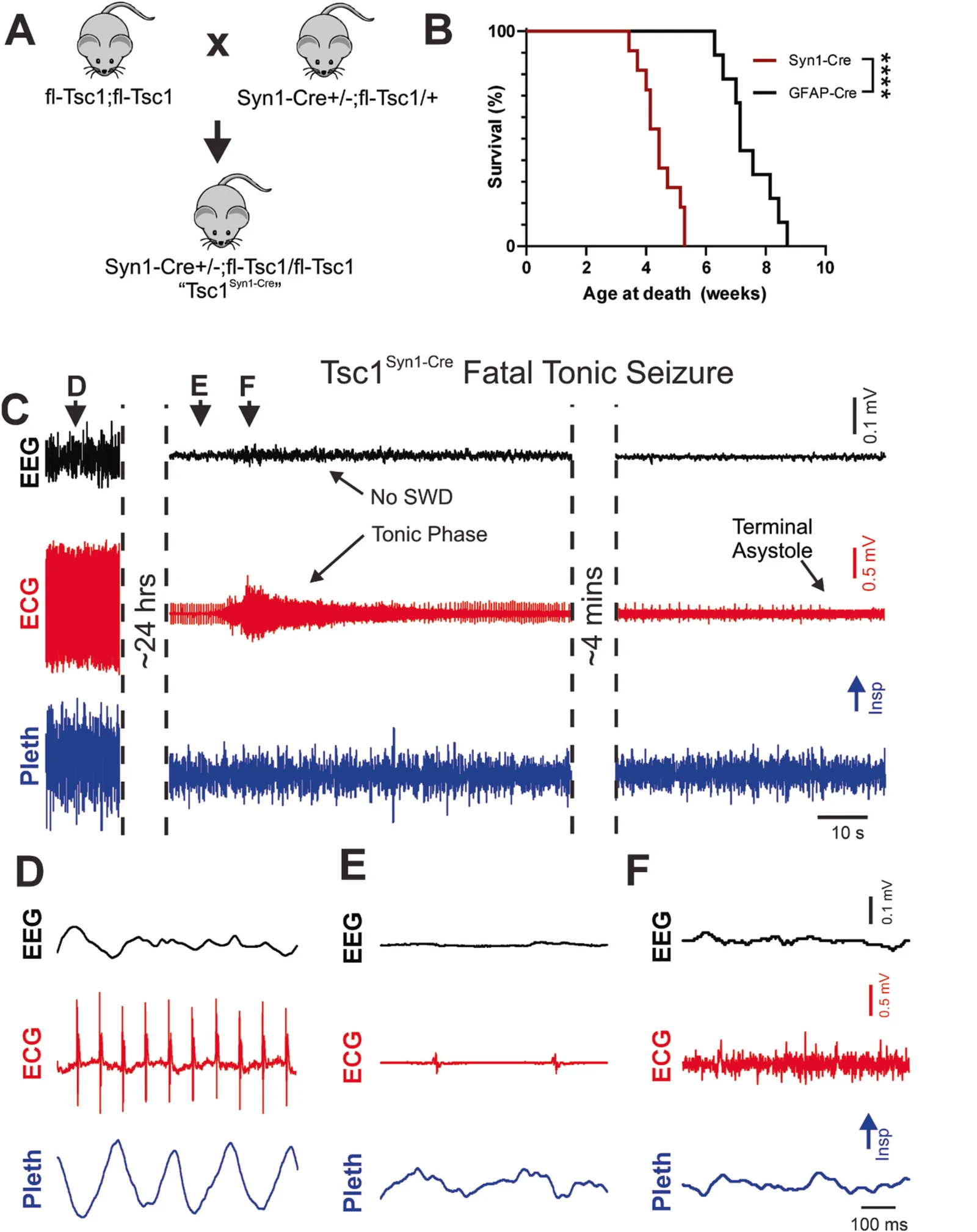
Tonic seizures are fatal in Tsc1^Syn1-Cre^ mice. A, Breeding strategy to produce TSC mouse model. B, Both TSC models experience early mortality and untreated Tsc1^GFAP-Cre^ mice live longer than Tsc1^Syn1-Cre^ mice (p < 0.0001, Mantel-Cox test; *n* = 9 and 11 for GFAP-Cre and Syn1-Cre mice, respectively). C, Example of EEG, ECG, and breathing (pleth) recording from a Tsc1^Syn1-Cre^ mouse 24 h before, during, and after a fatal seizure. D–F, Expanded signals from 24 h before (D), immediately before (E), and during (F) the tonic phase of the fatal seizures. Note that the breathing signal was clear in D but is degraded in E and F.

**Fig. 6.**
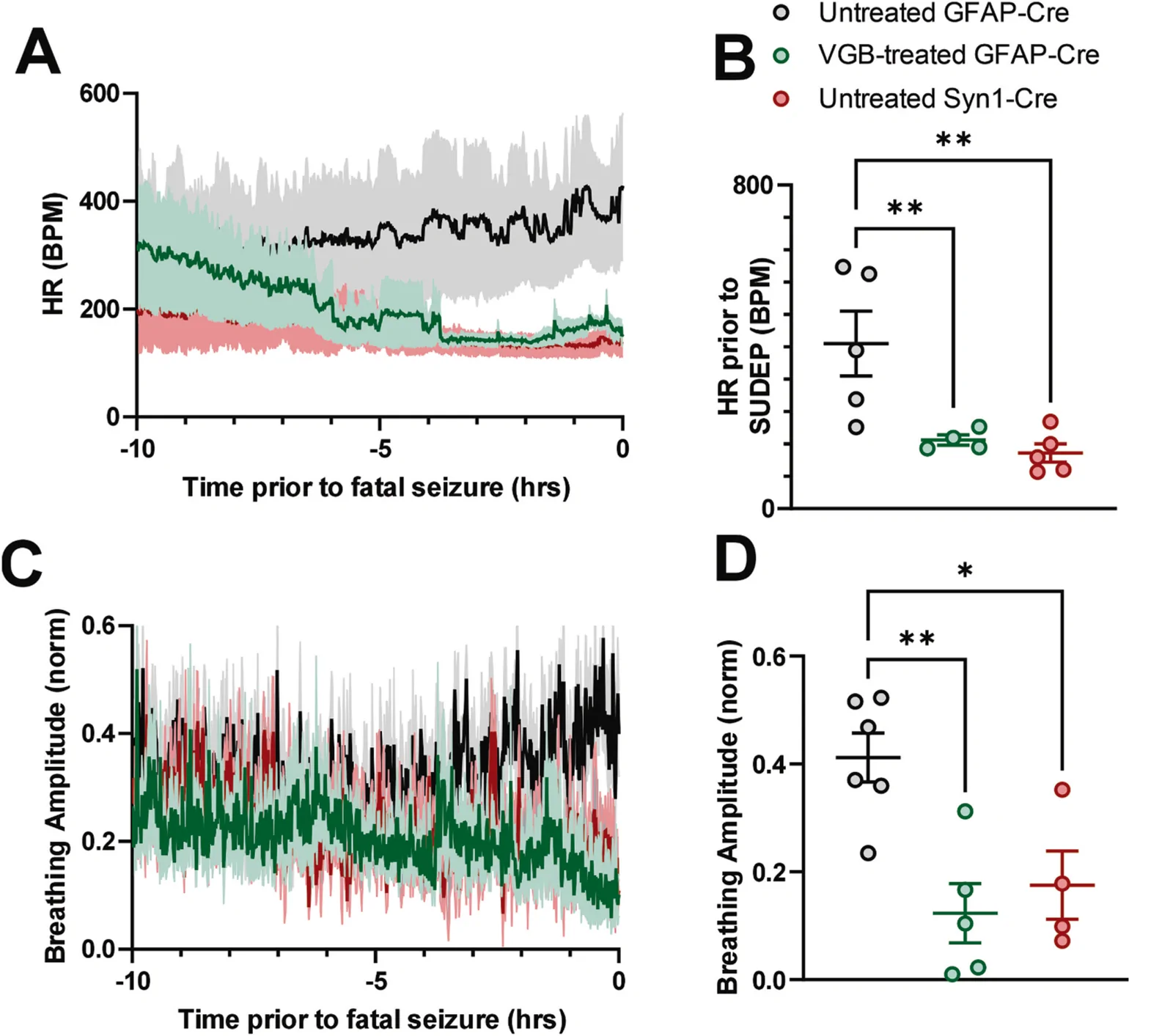
Breathing and heart rate (HR) declines prior to death for Tsc1^Syn1-Cre^ mice and Tsc1^GFAP-Cre^ mice treated with VBG, but not for control Tsc1^GFAP-Cre^ mice. A, HR (line = mean, shaded region = SEM) over the 10 h before fatal seizures for untreated Tsc1^GFAP-Cre^ mice (black), VGB-treated Tsc1^GFAP-Cre^ mice (green), and untreated Tsc1^Syn1-Cre^ mice (red). B, Mean HR during the one hour before fatal seizures for each group. The VGB-treated Tsc1^GFAP-Cre^ and untreated Tsc1^Syn1-Cre^ mice had significantly lower HR compared to untreated mice (*p* = 0.0091 and 0.0059, respectively after significant one-way ANOVA, F = 8.4 and *p* = 0.0061). C, Breathing amplitude (line = mean, shaded region = SEM) over the 10 h before fatal seizures for untreated Tsc1^GFAP-Cre^ mice (black), VGB-treated Tsc1^GFAP-Cre^ mice (green), and untreated Tsc1^Syn1-Cre^ mice (red). D, Mean breathing amplitude during the one hour before fatal seizures for each group. The VGB-treated Tsc1^GFAP-Cre^ and untreated Tsc1^Syn1-Cre^ mice had significantly lower HR compared to untreated mice (*p* = 0.0181 and 0.0034, respectively after significant one-way ANOVA, F = 9.1 and *p* = 0.0039). (For interpretation of the references to colour in this figure legend, the reader is referred to the web version of this article.)

## Data Availability

The data that support the findings of this study are available from the corresponding author upon reasonable request.

## Appendix A. Supplementary data

Supplementary data to this article can be found online at https://doi.org/10.1016/j.nbd.2026.107524.
