# Supplementary material for: Seizure-induced apnea causes sudden death independent of cortical spike wave discharge in mouse models of Tuberous Sclerosis Complex
Source: Neurobiol Dis. Author manuscript; Available in PMC 2026 Oct 8. (PMC13647238; doi:10.1016/j.nbd.2026.107524)
Supplement: Supplementary file 1 [file NIHMS2198301-supplement-MMC1.docx]

# Supplemental Figures

**Supplementary Figure 1. A,** EEG trace during a clonic seizure of a Tsc1^GFAP-Cre^ mouse. **B,** EEG trace during a tonic seizure of a Tsc1^GFAP-Cre^ mouse. **C,** Power spectrum from 0 to 50 Hz from a 20 s preictal (black) and 20 s ictal (purple) of a clonic seizure. **D,** Power spectrum from 0 to 50 Hz from a 20 s preictal (black) and 20 s ictal (purple) of a tonic seizure. Inset, expanded y-axis from D showing right-shifted peak frequency for ictal period (purple).

**Supplemental Figure 2.** **A,** EEG, ECG, and breathing (pleth) recording during a fatal, non-tonic seizure from a single Tsc1^GFAP-Cre^ mouse. As with fatal tonic seizures, terminal apnea preceded terminal asystole. **B,** Expanded region from A of terminal apnea initiation demonstrates a lack of tonic muscle contraction. In addition, no hindlimb extension was observed in the video (data not shown).

**Supplemental Figure 3.** **A,** All recorded fatalities in Tsc1^Syn1-Cre^ mice had terminal apnea that preceded terminal asystole. **B,** No significant difference was detected between Tsc1^GFAP-Cre^ and Tsc1^Syn1-Cre^ mice.
